# Selection Index in the Study of Adaptability and Stability in Maize

**DOI:** 10.1155/2014/360570

**Published:** 2014-02-13

**Authors:** Rogério Lunezzo de Oliveira, Renzo Garcia Von Pinho, Daniel Furtado Ferreira, Luiz Paulo Miranda Pires, Wagner Mateus Costa Melo

**Affiliations:** ^1^Engenheiro Agrônomo, Mestre em Genética e Melhoramento de Plantas, DuPont do Brasil S.A. Divisão Pioneer Sementes, Rodovia GO 210 km 04, Fazenda Santa Maria de Baixo, Zona Rural, Caixa Postal 1014, 75503-970 Itumbiara, GO, Brazil; ^2^Engenheiro Agrônomo, Doutor em Genética e Melhoramento de Plantas, Departamento de Agricultura/DAG, Universidade Federal de Lavras (UFLA), Lavras, MG, Brazil; ^3^Engenheiro Agrônomo, Pós-Doutor em Estatística e Experimentação Agronômica, Departamento de Ciências Exatas (DEX), Universidade Federal de Lavras (UFLA), Lavras, MG, Brazil; ^4^Engenheiro Agrônomo, Mestre em Genética e Melhoramento de Plantas, Departamento de Biologia/DBI, Universidade Federal de Lavras (UFLA), Lavras, MG, Brazil; ^5^Engenheiro Agrônomo, Doutor Agronomia/Fitotecnia, Advanta Comércio de Sementes Ltda, Florianópolis, SC, Brazil

## Abstract

This paper proposes an alternative method for evaluating the stability and adaptability of maize hybrids using a genotype-ideotype distance index (GIDI) for selection. Data from seven variables were used, obtained through evaluation of 25 maize hybrids at six sites in southern Brazil. The GIDI was estimated by means of the generalized Mahalanobis distance for each plot of the test. We then proceeded to GGE biplot analysis in order to compare the predictive accuracy of the GGE models and the grouping of environments and to select the best five hybrids. The G × E interaction was significant for both variables assessed. The GGE model with two principal components obtained a predictive accuracy (PRECORR) of 0.8913 for the GIDI and 0.8709 for yield (t ha^−1^). Two groups of environments were obtained upon analyzing the GIDI, whereas all the environments remained in the same group upon analyzing yield. Coincidence occurred in only two hybrids considering evaluation of the two features. The GIDI assessment provided for selection of hybrids that combine adaptability and stability in most of the variables assessed, making its use more highly recommended than analyzing each variable separately. Not all the higher-yielding hybrids were the best in the other variables assessed.

## 1. Introduction

In breeding programs, it is often necessary to obtain measures of various traits to select them simultaneously. This need led to the development of selection indices, allowing selection to be performed using a single value. The application of selection indices was initially proposed by Smith [1] through the optimized index. This index was later modified, and most of these modifications were also based on linear combinations of the phenotypic values observed. The most common modifications are those of Brim et al. [2], Kempthorne and Nordskog [3], Pešek and Baker [4], and Tai [5] and Smith et al. [6].

Other authors presented nonparametric indices, applied for the purpose of classifying genotypes. Elston [7] proposed a multiplicative index which considers all the traits with the same economic weight. Wricke and Weber [8] suggested the use of the Euclidean and Mahalanobis distances to classify the genotypes for various traits simultaneously, based on their distance from an ideal genotype (ideotype), defined by the researcher. Mulamba and Mock [9] developed a quite simple index, which uses the sum of the number of order that the genotype presents for each trait; the lower the value obtained in this sum, the better the classification.

There are various methodologies for evaluation of the G × E interaction. Crossa [10] suggests that the application of multivariate methods may be useful to better exploit the information contained in the data. Recently, a modification of the conventional AMMI analysis proposed by Yan et al. [11], called the GGE biplot, has been used for study of the G × E interaction. GGE analysis groups the genotype effect, which is an additive effect in the AMMI analysis, with the G × E interaction, which is a multiplicative effect, subjecting these effects to a multiplicative model of regression for sites (SREG - Site Regression). The main advantage of this technique in relation to AMMI analysis resides in the fact that the GGE biplot method always explains an intermediate portion of the sum of squares of genotypes (G) + interaction (G × E) in relation to the AMMI1 (with one principal component) and AMMI2 (with two principal components) models, Yan et al. [12].

Garcia and Júnior [13] and Farshadfar [14] incorporated stability and adaptability parameters in selection indices to select individuals of greatest stability and best performance. Nevertheless, these studies that applied nonparametric selection indices as parameters of incorporated stability did it with the mean values of the cultivars in all the environments.

It is difficult to find studies in the literature that have analyzed these selection indices at plot level and have analyzed the use of these indices for adaptability and stability analyses by multivariate methods such as the GGE biplot so that the applicability of a nonparametric selection index may be better evaluated and utilized. For that reason, the aim of this study was to evaluate the adaptability and stability of the genotype-ideotype distance index and selection of the best hybrids by means of this alternative.

## 2. Materials and Methods

Assessment data were composed of seven traits from 25 maize hybrids subjected to performance evaluation trials in six locations of the south of Brazil (Vacaria, RS; Abelardo Luz, SC; Candoi, PR; Canoinhas, SC; Castro, PR; and Ponta Grossa, PR) during the 2010/2011 crop year. The trials were prepared in a completely randomized block design with two replications. The plots were composed of four five-meter rows, with a spacing of 70 cm between rows. Fertilizations were carried out following the recommendations made for each location by means of soil analysis. Crop treatments necessary for control of army worm (*Spodoptera frugiperda*), corn earworm (*Helicoverpa *zea), and weeds were carried out.

The traits assessed were grain yield—kg ha^−1^, percentage of damaged grains, percentage of lodging, percentage of breakage, percentage of fallen plants, common rust (*Puccinia sorghi*) score, and gray leaf spot (*Cercospora zeae-maydis*) score.

The percentage of damaged grains was determined through the proportion of grains with symptoms of rotting in a 1000 grain sample. The percentage of lodging was calculated through the proportion of plants at an inclination of more than 30° due to weakening of the roots in relation to the total number of plants in the plot. The percentage of fallen plants, for its part, corresponded to the proportion of plants at an inclination of more than 30° due to weakening of the base of the stalk in relation to the total number of plants in the plot. The disease severity score was based on the diagrammatic scale proposed by Agroceres [15], which ranges from 1 to 9, in which score 1 represents a leaf without symptoms and score 9 corresponds to the material with more than 80% of the leaf area affected.

The ideotype for yield (kg ha^−1^) was determined seeking the value of the highest yielding plot of all the trails and using the next thousand value above that. As the highest yielding plot was 16959 kg ha^−1^, the ideotype was 17000 kg ha^−1^. For the traits of percentage of damaged grains, percentage of lodging, percentage of fallen plants, and percentage of breakage, the ideotype was 0%. For common rust and grey leaf spot, the ideotype was score 1.

To obtain the matrix of variances and covariances among the traits assessed, multivariate analysis of variance (MANOVA) was performed using the data from all the hybrids, traits, and locations. For that purpose, the model according to Ferreira [16] was used, expressed in the vectorial form in the following expression:

(1) $$\begin{array}{c} Y_{ijk}=\;\mu +\alpha_{i}+\;\beta_{i}+\delta_{ij}+\varepsilon_{ijk}, \end{array}$$

in which the vector *Y*_*ijk*_ = [*Y*_*ijk*1_, *Y*_*ijk*2_,…, *Y*_*ijkp*_] refers to the multivariate observations associated with the *i*th hybrid (*i* = 1, 2,…, 25), at the *j*th location (*j* = 1, 2,…, 6) in the *k*th replication of this combination of the *i* and *j* levels of the two factors; *μ* is the vector of constants of the multivariate linear model given by *μ*_*i*_ = [*μ*_*i*1_, *μ*_*i*2_,…, *μ*_*ip*_], *α*_*i*_ is the vector of effects of the *i*th hybrid given by *α*_*i*_ = [*α*_*i*1_, *α*_*i*2_,…, *α*_*ip*_], *β*_*j*_ is the vector of effects of the *j*th location given by *β*_*j*_ = [*β*_*j*1_, *β*_*j*2_,…, *β*_*jp*_], *δ*_*ij*_ is the vector of effects of the interaction between the *i*th hybrid and the *j*th location given by *δ*_*ij*_ = [*δ*_*i*1_, *δ*_*i*2_,…, *δ*_*ip*_], and *ε*_*ijk*_ is the vector of effects of the nonobservable experimental error corresponding to observation *Y*_*ijk*_. The manova package of the software R v 3.0 [17] was used for MANOVA.

The genotype-ideotype distance index (GIDI) for selection was obtained using the data at the plot level of the seven traits assessed in the 25 hybrids. Thus, for each plot under assessment, an index was obtained based on the generalized Mahalanobis distance, using the matrix model according to the following expression:

(2) $$\begin{array}{c} D_{Gl}^{\;2}=\mathrm{diag}\left({IS}^{-1}I^{\prime }\right), \end{array}$$

where the term *IS* ^−1^ *I*′ is written in the following manner:

(3) |(Y1111−Y1ℓ)⋯(Yn111−Ynℓ)⋮⋱⋮(Y1her−Y1ℓ)⋯(Ynher−Ynℓ)|(N×n) ×|V1⋯COV1n⋮⋱⋮COVn1⋯Vn|(n×n)−1 ×|(Y1111−Y1ℓ)⋯(Y1her−Ynℓ)⋮⋱⋮(Yn111−Y1ℓ)⋯(Ynher−Ynℓ)|(n×N)

in which *D*_*Gℓ*_^2^ is the matrix of the distances of *h*  (*h* = 1, 2,…, 25) hybrids in relation to the ideotype *ℓ*; *Y*_*ijkl*_; is the observation of trait *i*  (*i* = 1, 2,…, *n*) where *n* = 7, of hybrid *j*  (*j* = 1, 2,…, *h*), at location *k*  (*k* = 1, 2,…, *e*) where *e* = 6, in replication *l*  (*l* = 1, 2); *V*_1_, *V*_2_,…, *V*_*n*_ are the variances of the residues (QMerro of the multivariate analysis of variance) of the *n* traits assessed and COV_12_, COV_2*n*_,…, COV_(*n*−1)*n*_ are the covariances among the residues of these traits.

After obtaining the GIDI for each plot, joint analysis of variance was performed to verify the presence of the G × E interaction for the GIDI and for grain yield. Once the presence of the G × E interaction (*F* test significant) was observed, analysis of adaptability and stability was performed for the two variables, which allowed the adaptation and stability of each hybrid under testing to be measured, for the GIDI and for yield. This evaluation was made using the GGE biplot method. As the interpretation of the GIDI is the opposite of yield, that is, the lower the value of the index, the better the performance, the value of each plot was subtracted from 2000. This value was determined using the same principle used in the determination of ideotype of yield, that is, using the next thousand value above the greatest value of the index considering all the plots of all the trails. Graph interpretation was thereby able to be performed in the same manner as yield. GGE biplot analysis was performed by means of the package GGEBiplotGUI of the software R v 3.0 [17].

Analysis by means of the GGE biplot method was performed as presented by de Oliveira et al. [18], considering the simplified model for two principal components:

(4) $$\begin{array}{c} {\overset{-}{Y}}_{ij}-\mu_{j}=\lambda_{1}\gamma_{i1}\alpha_{j1}+\lambda_{2}\gamma_{i2}\alpha_{j2}+\rho_{ij}+{\overset{-}{\varepsilon }}_{ij}, \end{array}$$

in which ${\overset{-}{Y}}_{ij}$ is the mean value of hybrid *i* in location *j*, for the GIDI or yield; *μ*_*j*_ is the mean value of the location *j*;  *λ*_1_ *γ*_*i*1_ *α*_*j*1_is the first principal component (PCA 1) of the effect of genotypes (G) + interaction (G × A); *λ*_2_ *γ*_*i*2_ *α*_*j*2_ is the second principal component (PCA 2) of the effect of genotypes (G) + interaction (G × A); *λ*_1_ and *λ*_2_ are the eigenvalues associated with the PCA 1 and with the PCA 2; *γ*_*i*1_ and *γ*_*i*2_ are the scores of the PCA 1 and of the PCA 2, respectively, for genotypes; *α*_*j*1_ and *α*_*j*2_ are the scores of the PCA 1 and of the PCA 2, respectively, for environments; *ρ*_*ij*_ is the residue of the genotype × environment interaction, also known as “noise”, corresponding to the principal components not retained in the model; ${\overset{-}{\varepsilon }}_{ij}$ is the residual of the model with normal distribution, mean value zero, and variance *σ* ^2^/*r* (where *σ* ^2^ is the variance of the error between plots for each environment and *r* is the number of replications).

The graph accuracy of the identification methods of megaenvironments and winning genotypes was tested by the cross validation procedure proposed by Gabriel [19]. For that purpose, the PRESSm and PRESScorr statistics were used to measure the discrepancy between the observed and predicted values and the predictive correlation [20]. This cross validation analysis was performed by means of PROC IML of the statistical package of the software SAS v 9.0 [21].

The GGE biplot graph was constructed as of the data from breakdown of the empirical mean values, graphically presenting the hybrid with the best development, as detailed by de Oliveira et al. [18]. The environments were grouped by means of the graphic approach called “Which Won Where” by Yan et al. [11]. Thus, the environments that share the same winning genotype remain in the same group.

The five hybrids with greatest adaptability and stability for the GIDI and yield were identified and selected by means of their distance in relation to the “ideal genotype” which was plotted in the center of a “target” in the biplot. That way, the hybrids plotted nearer the center of this “target” are those that have the best combination between adaptability and stability as presented by Balestre et al. [22].

## 3. Results and Discussion

The G × E interaction was significant (*P* ≤ 0.01), showing that the performance of the hybrids was not consistent in the different assessment environments in regard to the GIDI (data not shown). The results of analysis of variance for grain yield were similar to those obtained in the GIDI analysis. The genotype and environment sources of variation and their interaction were significant (*P* ≤ 0.01). The coefficient of variation was very low (3.3%), which shows the high experimental precision of the trails.

The mean value of the hybrids for grain yield (kg ha^−1^) and for the GIDI (already subtracted from 2000, to make its interpretation similar to that of yield), involving the six locations, are shown in Table 1.

As may be observed, the highest yielding hybrids are not necessarily those that have the greatest values of the GIDI. This is due to the fact that the highest yielding hybrids are not always the best in the other traits assessed and considered in the GIDI.

The results of cross validation of the GGE biplot model for the GIDI and for grain yield are shown in Table 2.

In this study, only the results of cross validation for the first two principal components were presented since the reduced model was chosen due to the difficulty of evaluating the biplot in more than two dimensions.

It may be seen that for the GGE2 model, the predictive accuracy (PRECORR_(m)_) was high for the two variables analyzed. In addition, the values were close when the two variables are compared. de Oliveira et al. [18] and Balestre et al. [22], evaluating the stability and adaptability of grain yield in maize and rice, obtained a PRECORR of 0.8617 and 0.90, respectively. These values are very near those obtained in this study. The GGE2 model was therefore accurate for the two variables.

**Figure 1:**
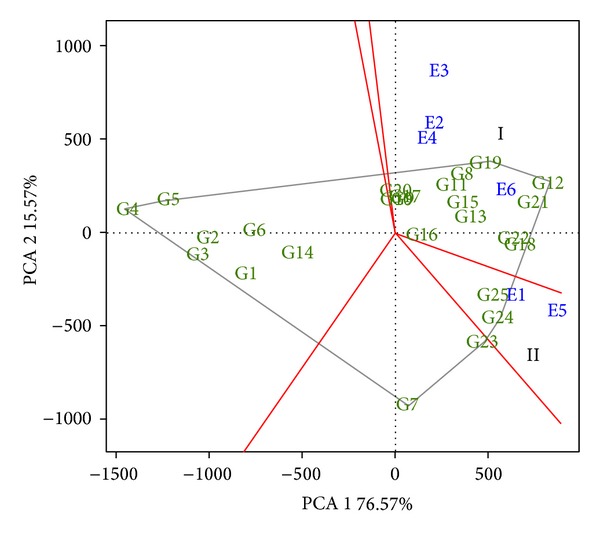
GGE biplot graph, evaluating the GIDI, with the grouping of the six locations (Vacaria, RS; Abelardo Luz, SC; Candoi, PR; Canoinhas, SC; Castro, PR; and Ponta Grossa, PR) identified from E1 to E6, respectively, with the groups of environments being identified by the Roman numerals I and II; the gray line represents the polygon formed by genotypes evaluated.

Subsequently, the biplot was obtained and the “Which Won Where” approach was carried out to identify the environments or megaenvironments, evaluating the GIDI (Figure 1). In this figure, the hybrids were identified from G1 to G25.

**Figure 2:**
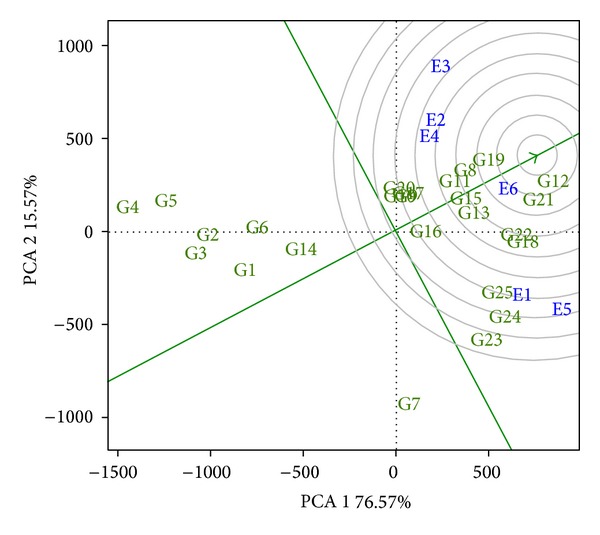
GGE biplot graph, with the distance of the 25 hybrids (identified from G1 to G25) from the “ideal genotype” in regard to the GIDI. Concentric circles facilitate the exploratory analysis of the distances between the genotypes evaluated and the ideal genotype represented by the green arrow.

The first result to be highlighted is the high explanatory power of the sum of squares of G + G × E presented by the two principal components of the biplot (PCA 1 and PCA 2). The two components added together accounted for 92.14%, showing that the analysis was very efficient. In regard to grouping, two groups were obtained, identified on the graph by the Roman numerals I and II. Group I was composed only of locations 2, 3, 4, and 6 (Abelardo Luz, SC; Candoi, PR; Canoinhas, SC; and Ponta Grossa, PR; resp.) in which hybrid 12 was the winner. Group II was composed of locations 1 and 5 (Vacaria, RS and Castro, PR resp.) in which hybrid 24 was the winner. In this analysis, we can also observe that location 6 (Ponta Grossa, PR) was that which presented the greatest ability for discriminating genotypes due to its high score in the first principal component. Evaluation of adaptability and stability of the hybrids by means of the approach of distance from the “ideal genotype” was also performed (Figure 2).

It may be observed that hybrids 12, 21, 19, 8, and 22 are the nearest to the “ideal genotype” in that order. Therefore, they are the five best hybrids combining adaptability and stability.

**Figure 3:**
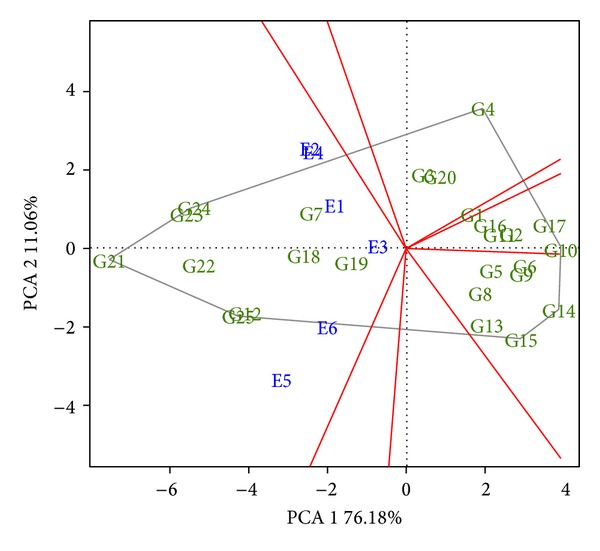
GGE biplot graph, evaluating grain yield (t ha^−1^), with the grouping of the six locations (Vacaria, RS; Abelardo Luz, SC; Candoi, PR; Canoinhas, SC; Castro, PR; and Ponta Grossa, PR), identified from E1 to E6, respectively, the gray line represents the polygon formed by genotypes evaluated.

GGE biplot analysis was also performed for the yield data, transformed into ton ha^−1^ to improve the scale of the principal components of the biplot. In relation to the portion of the sum of squares of G + G × E explained by the two principal components (Figure 3), this also was high (87.24%); however, a little less than that found in the analysis using the GIDI (92.14%). Likewise, the “Which Won Where” approach was performed by means of which it was seen that all the locations were in the same group (Figure 3).

Nevertheless, in analysis with the GIDI, two groups were identified. This difference is due to the fact of the GIDI aggregating other variables, not only grain yield, which makes the G × E interaction more complex when compared to the analysis only with yield, such that the use of the GIDI in grouping of environments becomes more informative.

**Figure 4:**
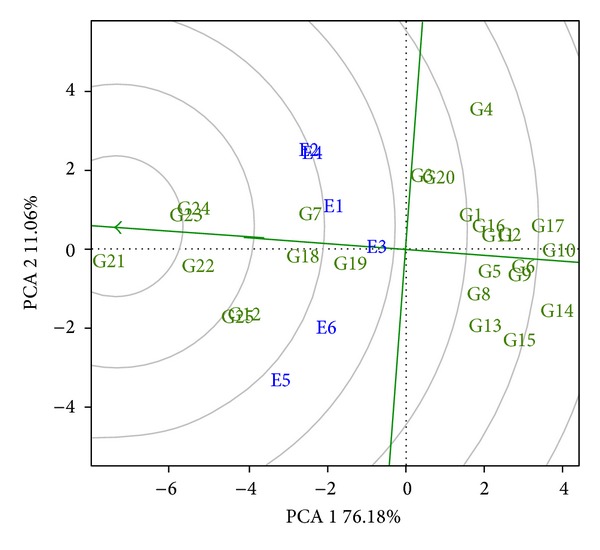
GGE biplot graph, with the distance of the 25 hybrids (numbered from G1 to G25) from the “ideal genotype”, in regard to grain yield (t ha^−1^). Concentric circles facilitate the exploratory analysis of the distances between the genotypes evaluated and the ideal genotype represented by the green arrow.

Subsequently, evaluation of the adaptability and stability of the hybrids for grain yield was performed by means of their distances from the “ideal genotype” (Figure 4).

The five hybrids nearest to the “ideal genotype” were hybrids 21, 23, 24, 22, and 25, in that order. Comparing this selection with that obtained by the analysis with the GIDI (hybrids 12, 21, 19, 8, and 22), it may be seen that only hybrids 21 and 22 were common to the two selections. There was also a change in the position of hybrids 21 and 22 comparing the two selections. To diagnose the reason for this difference, the measurements of each hybrid were obtained for each one of the seven variables assessed considering the six locations.

Garcia and Júnior [13] also used the GIDI estimated by means of the Euclidean distance for selection of maize hybrids and, to this, they incorporated the parameters of adaptability and stability, *b* and *R* ^2^, respectively. This procedure led to the selection of stable and adapted hybrids only for year yield (kg ha^−1^). Euclidean distance may only be applied to traits which are mutually independent, which was not the case of the traits used by the authors, with a negative effect on the selection obtained by them. For that reason, these authors recommended that the correlation between the variables assessed must be considered in the selection index. This correction was performed in this study since the GIDI was estimated by means of the Mahalanobis distance, which is weighted by the matrix of variances and covariances. In addition, the evaluation of adaptability and stability was performed with the GIDI estimated at the plot level, allowing the selection of adapted and stable hybrids for all the variables analyzed.

## 4. Conclusion

The evaluation of adaptability and stability of the GIDI led to the selection of hybrids that combine adaptability and stability for most of the traits assessed. Use of it is more practical than analyzing each trait separately.

## Figures and Tables

**Table 1:** Classification in decreasing order of the hybrids in regard to their mean grain yield (kg ha^−1^) and their respective mean values of the GIDI considering the six assessment locations.

Hybrids	Yield (kg ha^−1^)	GIDI
21	14713.84	1784.55
23	13923.10	1530.36
24	13903.24	1576.26
22	13844.71	1702.09
12	13166.87	1826.48
25	13146.49	1605.71
18	12589.40	1706.14
7	12448.18	1331.71
19	12347.06	1733.43
3	11637.13	1067.36
20	11612.62	1534.22
8	11210.67	1654.61
4	11013.32	997.00
1	10999.73	1159.46
11	10984.10	1632.94
16	10956.94	1528.95
13	10870.05	1630.95
5	10850.68	1049.96
15	10636.55	1621.16
2	10603.71	1118.82
9	10563.95	1519.58
6	10558.86	1192.99
17	10334.76	1532.99
10	10280.67	1510.38
14	10059.97	1258.74

**Table 2:** Estimates obtained for PRESS_m_ (sum of squared prediction error) and PRECORR_(m)_ (predictive accuracy) in cross validation of the GGE1 and GGE2 models (biplot with 1 and 2 principal components, resp.) for the GIDI and for yield (ton ha^−1^).

Model	GIDI		Yield	
	PRESS_m_	PRECORR_(m)_	PRESS_m_	PRECORR_(m)_
GGE1	36324.678	0.8086993	1.0547498	0.8656868
GGE2	21597.326	0.8913184	1.0179156	0.8708956
